# Interactions among *IGF-1*, *AKT2*, *FOXO1*, and *FOXO3* variations and between genes and physical activities on physical performance in community-dwelling elders

**DOI:** 10.1371/journal.pone.0239530

**Published:** 2020-09-28

**Authors:** Tsai-Chung Li, Ching-Wei Wu, Chia-Ing Li, Fang-Yang Wu, Li-Na Liao, Chiu-Shong Liu, Chih-Hsueh Lin, Mu-Cyun Wang, Chuan-Wei Yang, Cheng-Chieh Lin

**Affiliations:** 1 Department of Public Health, College of Public Health, China Medical University, Taichung, Taiwan; 2 Department of Healthcare Administration, College of Medical and Health Sciences, Asia University, Taichung, Taiwan; 3 School of Medicine, College of Medicine, China Medical University, Taichung, Taiwan; 4 Department of Medical Research, China Medical University Hospital, Taichung, Taiwan; 5 Department of Family Medicine, China Medical University Hospital, Taichung, Taiwan; Ehime University Graduate School of Medicine, JAPAN

## Abstract

This study assessed the interactions among *IGF-1*, *AKT2*, *FOXO1*, and *FOXO3* variations and the interactions of gene and physical activity on handgrip strength, arm muscle mass-adjusted handgrip (armGrip), gait speed (GS), timed up and go (TUG), and leg press strength (LPS). Nine single nucleotide polymorphisms (SNPs) containing three *IGF-1* SNPs (rs6214, rs5742692, and rs35767), two *AKT2* SNPs (rs892119 and rs35817154), two *FOXO1* SNPs (rs17446593 and rs10507486), and two *FOXO3* SNPs (rs9480865 and rs2153960) were genotyped in 472 unrelated elders with a mean age of 73.8 years. We observed significant interactions of *IGF-1* SNP rs6214 and rs35767 with regular physical activity on TUG and GS; and *AKT2* SNP rs892119 and *FOXO3* SNP rs9480865 with regular physical activity on armGrip. Genotype GG of *IGF-1* rs6214 and rs35767 in individuals without regular physical activity had poor performance in TUG and GS, as well as GG of *AKT2* rs892119 decreased armGrip in individuals without regular physical activity. After FDR adjustment, no significant gene–gene interactions were found. A sedentary lifestyle may increase the risk of impairing physical performance and regular physical activity is a remedy for sarcopenia, even a little regular physical activity can overcome carrying some risk alleles in this pathway.

## Introduction

The global population rapidly ages, and the life expectancy of older people continuously increases; the prevention or delay of age-associated mobility disability is an important public health issue [1]. Aging, even in healthy elderly individuals, is accompanied by a progressive decline in muscle mass and physical performance [2, 3]. Sarcopenia is defined as age-related loss of skeletal muscle mass, muscle strength, and physical performance [4–7] that is associated with adverse health outcomes and affects quality of life [8]. The European Working Group on Sarcopenia in Older People (EWGSOP) recommends that ‘physical performance should be considered a measure of the severity of sarcopenia’ [5]. There are several measurement tools to assess physical performance, such as handgrip strength, usual gait speed (GS), timed up and go test (TUG), etc. [5, 8–10]. The diagnosis of sarcopenia in clinical practice starts with the measurement of muscle strength, usually handgrip strength [5, 8]. Handgrip strength is a simple measurement parameter used to assess the overall muscle strength of elderly people in clinical settings [11]. It may serve as a predictor of health-related prognosis and is related to physical function performance, disability, and mortality [3, 12]. Previous studies showed that the change of muscle quality might be considered to precede the loss of muscle mass [13, 14], and muscle quality might explain to be a more relevant concept to health than muscle mass [8]. To assess the arm muscle quality, the arm muscle mass-adjusted handgrip (armGrip) was used in this study. Furthermore, lower extremity strength is important in providing a stable base for movement and performing activities of daily living. Lower extremity muscle strength, assessed by leg press strength (LPS), is utilized to predict function performance in elders with mobility disability [15]. LPS is a measure of bilateral leg extension exercise. Usual GS is the most frequently used test and is also a useful screening tool to identify older adults at risk of hospitalization, a decrease in functional ability and mortality [2, 3]. TUG is a parameter that can be used to predict the risk of falling [16] and short-term mortality [17] in community-dwelling elderly. The EWGSOP suggests using these two tests, GS and TUG, for assessment of physical performance [5].

The IGF1–AKT–FOXO pathway plays an important role in aging [18–20]. Protein degradation is identified as a major determinant of muscle atrophy and regulated by a conserved pathway composed of insulin-like growth factor-1 (IGF-1). Serine/threonine kinase (AKT) has a major role in this pathway because it controls protein degradation by repressing the activation of the transcription factor Forkhead box O (FOXO). Single nucleotide polymorphisms (SNPs) may affect the expression of *IGF-1*, *AKT*, and *FOXO*. One prior study pointed out that SNP rs35767 affects circulating IGF-1 levels, showing that white European adults without diabetes who carried GG genotype of the rs35767 polymorphism, which is located in *IGF-1* promoter region, were associated with lower IGF-1 level compared with A allele carriers [21]. Individuals carrying homozygous genotypes of *IGF-1* SNPs (rs6214 and rs5742692) had significant higher IGF-1 levels comparing to reference genotypes [22]. Low IGF-1 levels in older women are associated with slow walking speed, poor muscle strength, and difficulty in performing mobility tasks [23]. The phosphatidylinositol-3-kinases (PI3K)/Akt pathway modulated by IGF-1 and insulin would control the muscle size [24]. *AKT2* becomes genetically disrupted and induces skeletal muscle atrophy in the mouse experiment [25]. Previous studies showed that insulin sensitivity is linked to skeletal muscle mass and sarcopenia in human research [26–29] or animal experiments [30]. It has been shown that the *AKT2* SNP rs892119 results in the activation of AKT2 [31]. The variation of rs35817154, missense variant in *AKT2* (R208K), has been observed among individuals with severe insulin resistance [32]. Evidence has shown that genetic variants (rs17446593 and rs10507486) in *FOXO1* gene were associated with β-cell dysfunction [33] or type 2 diabetes [34]. In vivo transgenic and knockout models, the FOXO family has emerged as the main regulator of muscle atrophy. *FOXO1* and *FOXO3* are atrophy-related genes whose overexpression can reduce muscle mass [35]. Furthermore, SNP rs2153960 in the *FOXO3* gene is correlated with circulating IGF-1 concentration in the genomewide meta-analysis [36]. Based on previous studies, brain activity has a significant role in human aging and longevity [37]; *FOXO3* gene has an association with longevity [38], and its SNP rs9480865 was found that this variant was associated with brain parenchymal volume [39]. However, studies have yet to address how *AKT2*, *FOXO1*, and *FOXO3* genetic variations in this pathway affect the physical performance of the elderly.

Sarcopenia is a complex multifactorial condition. The onset and progression of sarcopenia involves lack of physical activity, loss of neuromuscular function, altered endocrine function, genetic influence, and poor diet nutritional status [4, 5, 24, 40–42]. Prior heritability-related studies have shown that skeletal muscle traits have high heritability, such as muscle strength ranging 30–85% and lean mass ranging 50–80% [43]. Furthermore, the IGF1–AKT–FOXO signaling pathway plays an important role in sarcopenia [8, 18]. Dent et al. published evidence-based clinical practice guidelines for sarcopenia and they pointed out that physical activity is strongly recommended for the primary treatment of sarcopenia [44]. However, the evidence for nutrition interventions is less consistent [44]. Therefore, exploring interactions among genes within the IGF1–AKT–FOXO signaling pathway, as well as the interactions between modifiable lifestyle factors, such as physical activity, and genetic factors are essential for identifying the population at risk. We hypothesized *IGF-1*, *AKT2*, *FOXO1*, and *FOXO3* variants influence physical performance measures of handgrip strength, armGrip, TUG, GS, and LPS in the elderly. Specifically, the present study investigated three *IGF-1* SNPs (rs6214, rs5742692, and rs35767), two *AKT2* SNPs (rs892119 and rs35817154), two *FOXO1* SNPs (rs17446593 and rs10507486), and two *FOXO3* SNPs (rs9480865 and rs2153960) to test whether these variants interacted with each and with physical activity for physical performance measures of handgrip strength, armGrip, TUG, GS, and LPS in a cross-sectional study involving community-dwelling elders in Taiwan.

## Methods

### Study subjects

This study consisted of 472 subjects (251 men and 221 women) aged 65 years or older (mean age 74.7 and 72.8 years for men and women, respectively) and participated in Taichung Community Health Study for Elders (TCHS-E). The details of our study design and participant recruitment were the same as those in our previously published paper [45, 46]. In brief, our participants were recruited in a community-based cross-sectional study named TCHS-E. A total of 3,997 elders aged ≥ 65 years old were on the list of the sampling frame of eight LIs (administrative neighborhoods) in the North District of Taichung City in June 2009. Among them, 2,750 eligible elders were invited to participate in a physical check-up program, and 1,247 ineligible elders were excluded. There were a total of 1,347 elders agreed to participate in TCHS-E study, but 475 of these elders were excluded because of missing data (n = 11) and refusing DXA examination (n = 464). Out of 872 participants of TCHS-E, 480 unrelated elders provided DNA were recruited as subjects. In this study, 472 elders were successfully genotyped. Each participant provided his/her written informed consent. This study was conducted after obtaining approval from the Institutional Review Board of China Medical University Hospital (DMR97-IRB-055) and all methods were performed in accordance with the relevant guidelines and regulations.

### Measurements

The participants completed a self-administered questionnaire with information on demographic and lifestyle characteristics, self-reported health status, and overall obesity measured by body mass index (BMI) calculated as weight divided by height squared (kg/m^2^). Elders who reported that they regularly engaged in leisure-time activities for at least 30 min once every week and lasting at least 6 months were classified as having regular physical activity. Smoking status was categorized as non-smokers and smokers. Non-smokers never smoked or had smoked less than 100 cigarettes during their lifetime; smokers currently or previously smoked at least 100 cigarettes during their lifetime. Individuals who self-reported the characteristic of alcohol drinking was classified into the group with this specific characteristic. Blood sample was collected from each participant in the morning after a 12 h overnight fasting. These samples were analyzed within 4 h of collection.

Performance measures included handgrip strength, armGrip, TUG, GS, and LPS. Isometric handgrip strength was assessed for each hand by using a dynamometer (TTM Dynamometer, Tsutsumi, Tokyo, Japan). For each hand, measurements were repeated three times and their average handgrip strength (kg) was calculated. The highest average value of the left or right hand was used as the participant’s handgrip strength. ArmGrip (kg/kg) was determined by dividing handgrip strength by arm muscle mass measured via dual energy X-ray absorptiometry system (GE-LUNAR DPX, Lunar Corporation, Madison, WI) [47, 48]. TUG (s) is a simple timed measure that quantifies functional mobility. Participants were instructed to stand up from a chair, walk 3 m, turn around, walk back, and sit down. Each participant performed three trials, and the shortest time elapsed was used for data analysis. GS was measured over the 5-meter distance at the subjects’ usual pace, and the time spent was recorded. Gait speed (m/s) was calculated by dividing the distance walked by the time spent in seconds. Each participant’s submaximal leg press strength was measured by a leg press machine (AURA G3-S70, Matrix Fitness System, USA). All of the elders were asked to push a weight or resistance away from them by using their legs to evaluate their overall lower body strength from the knee joint to the hip. Then, the LPS (%) was calculated by dividing the corresponding results by the weight of the participant. The performance score was derived from combining armGrip, TUG, GS, and LPS. For scoring of each performance measure, the gender-specific first, second, and third quartiles of the 872 TCHS-E participants were used as cut-off points. Each performance indicator was given 0 to 3 points based on these cut points, that is, 0 points indicated the worst performance and 3 points were the best. Then, the four indicators were added up, and the total performance score was between 0 and 12 points.

### SNP selection and genotyping

Nine SNPs containing three *IGF-1* SNPs (rs6214, rs5742692, and rs35767), two *AKT2* SNPs (rs892119 and rs35817154), two *FOXO1* SNPs (rs17446593 and rs10507486), and two *FOXO3* SNPs (rs9480865 and rs2153960) were selected for genotyping based on previous studies and the HapMap dataset (CHB population). Genomic DNA was isolated from peripheral blood leukocytes by using a commercially available kit (QIAamp DNA Blood Kit; Qiagen, Chatsworth, CA, USA). The purified DNA concentration was determined with a ND-2000c spectrophotometer (NanoDrop Technologies, Wilmington, DE, USA). All SNPs were genotyped with an Illumina VeraCode GoldenGate genotyping assay (Illumina, San Diego, CA, USA). To increase the success rate of genotyping, the risk of genotyping failure was evaluated in advance, such as DNA quality. After genotyping, the overall call rate of these nine SNPs was 99.25%.

### Statistical analysis

The demographic characteristics and health status of study subjects were examined. Data were presented as the mean±standard deviation (SD) for continuous variables, or as a number and percentage for categorical variables. Bivariate statistical methods (eg, two-sample t-test, Chi-square test, one-way analysis of variance) were used to explore data features. Furthermore, we evaluated the Hardy–Weinberg equilibrium (HWE) for each SNP using PLINK software. Pairwise Linkage disequilibrium (LD) among SNPs were quantified by correlation coefficient, *r*^*2*^, using Haploview software. The linear regression analyses were used to evaluate the associations between physical performance measures and SNPs, under genetic models, including genotypic, additive, dominant, and recessive models. The multivariate model was further adjusted for age, sex, BMI, physical activity, smoking status, and alcohol drinking. Then gene–gene or gene–physical activity interactions on the physical performance measures were assessed by the multiple linear regression analyses. Then, stratified analysis and interaction plots were presented for those analysis with significant interactions. The p-values using false discovery rate (FDR) approach, a linear step up adjustment, was reported for gene–gene and gene–physical activity by considering multiple-testing problems. These analyses were carried out using Statistical Analysis System v9.4 (SAS Institute Inc., Cary, NC, USA), Haploview (v4.2) [49] and PLINK (v1.07) (http://pngu.mgh.harvard.edu/purcell/plink) [50]. Level of significance was set at two-sided *p* value<0.05.

## Results

### Basic characteristics of study subjects

The demographic baseline characteristics and health status of study subjects are summarized in Table 1. The participants had a mean age of 73.8 years, and 46.8% of them were women. In physical performance, handgrip strength, armGrip, TUG, GS, and LPS were significantly lower in women than in men (P<0.05).

**Table 1:** Demographic characteristics and health status of study subjects.

Characteristic	All	Women	Men	p-value *
	(n = 472)	(n = 221)	(n = 251)	
Gender, n (%)		221 (46.8%)	251 (53.2%)	n.a.
Age (years)	73.80 ± 6.07	72.78 ± 5.46	74.7 ± 6.45	0.001
BMI (kg/m^2^)	23.51 ± 3.24	23.48 ± 3.33	23.54 ± 3.18	0.523
**Physical performance**				
Handgrip strength (kg)	27.55 ± 8.53	20.89 ± 5.41	33.42 ± 6.14	<0.001
armGrip (kg/kg)	6.35 ± 1.25	6.07 ± 1.45	6.59 ± 0.99	<0.001
Timed up and go test (s)	7.94 ± 4.77	7.99 ± 4.79	7.90 ± 4.76	0.036
Gait speed (m/s)	0.85 ± 0.22	0.84 ± 0.21	0.87 ± 0.23	0.039
Leg press strength (%)	93.93 ± 38.10	82.64 ± 36.01	103.65 ± 37.22	<0.001
Performance score	6.67 ± 3.23	6.57 ± 3.27	6.77 ± 3.18	0.532
**Health indicators**				
Regular physical activity (%)	376 (79.66)	167 (75.57)	209 (83.27)	0.038
Alcohol drinking (%)	94 (19.92)	14 (6.33)	80 (31.87)	<0.001
Smoker (%)	100 (21.19)	7 (3.17)	93 (37.05)	<0.001
**Disease history**				
Hypertension	221 (47.42)	98 (45.16)	123 (49.40)	0.361
Diabetes	65 (13.92)	21 (9.55)	44 (17.81)	0.010
Hyperlipidemia	118 (25.65)	70 (32.11)	48 (19.83)	0.003
CVA	21 (4.56)	10 (4.59)	11 (4.53)	0.975
Cancer	25 (5.53)	13 (6.10)	12 (5.02)	0.615

Data were presented as mean±SD for continuous variables or n (%) for categorical variables.

armGrip: arms muscle mass-adjusted handgrip; BMI: body mass index; CVA: cerebral vascular accident.

*: Two-sample t test for continuous variables or chi-square test for categorical variables.

n.a.: not applicable.

### Genotype and allele frequencies in the four gene polymorphisms and their association with handgrip strength, armGrip, TUG, GS, and LPS

Table 2 reveals the corresponding descriptive statistics of genotype and allele distributions and physical performance based on various genotypes. Two SNPs at *AKT2* rs35817154 and *FOXO1* rs10507486 deviated from the HWE, which were excluded. TUG increased through the genotypes of *FOXO1* rs17446593 polymorphisms, and the number of minor alleles increased. LPS in individuals carrying AG variant of *FOXO3* rs2153960 also decreased compared with that in individuals carrying the GG and AA genotypes (P<0.05). In this study, the modest or weak degree of LD between the analyzed polymorphisms was detected (*r*^*2*^ = 0.48 between rs5742692 and rs35767; *r*^*2*^ = 0.41 between rs6214 and rs5742692; *r*^*2*^ = 0.16 between rs6214 and rs35767; and *r*^*2*^ = 0.12 between rs9480865 and rs2153960).

**Table 2:** Genotype and allele distributions of study subjects and their handgrip strength, armGrip, TUG, GS, LPS, and performance score distributions based on genotype status^a^.

SNP	Genotype or allele n (%)		Handgrip strength (kg)	armGrip (kg/kg)	TUG (s)	GS (m/s)	LPS (%)	Performance score
***IGF-1***								
rs6214	G G	125 (26.5)	26.99 ± 8.24	6.43 ± 1.27	8.68 ± 6.20	0.82 ± 0.25	90.21 ± 35.80	6.44 ± 3.43
	A G	235 (49.8)	27.58 ± 8.78	6.27 ± 1.27	7.75 ± 4.42	0.86 ± 0.21	95.35 ± 38.11	6.69 ± 3.19
	A A	112 (23.7)	28.12 ± 8.37	6.41 ± 1.21	7.48 ± 3.31	0.88 ± 0.19	95.08 ± 40.53	6.86 ± 3.08
	A*	459 (48.6)						
rs5742692	A A	241 (51.1)	27.43 ± 8.63	6.34 ± 1.28	8.28 ± 5.49	0.84 ± 0.23	92.17 ± 36.04	6.44 ± 3.35
	A G	188 (39.8)	27.85 ± 8.53	6.33 ± 1.18	7.69 ± 4.15	0.87 ± 0.21	96.80 ± 40.24	6.82 ± 3.13
	G G	41 (8.7)	27.14 ± 8.27	6.46 ± 1.44	7.19 ± 2.09	0.88 ± 0.19	90.84 ± 38.95	7.23 ± 2.91
	G*	270 (28.7)						
rs35767	G G	184 (39.0)	27.80 ± 8.37	6.38 ± 1.19	8.02 ± 5.16	0.86 ± 0.23	95.01 ± 36.92	6.76 ± 3.35
	A G	219 (46.4)	27.33 ± 8.95	6.25 ± 1.35	7.88 ± 4.49	0.84 ± 0.21	94.75 ± 39.74	6.46 ± 3.20
	A A	55 (11.7)	27.59 ± 7.35	6.55 ± 1.14	7.92 ± 4.17	0.87 ± 0.19	88.64 ± 36.21	6.98 ± 2.92
	A*	329 (35.9)						
***AKT2***								
rs892119	G G	362 (76.7)	27.34 ± 8.63	6.30 ± 1.30	8.06 ± 5.18	0.86 ± 0.23	93.69 ± 38.43	6.61 ± 3.27
	A G	94 (19.9)	27.96 ± 7.78	6.54 ± 1.10	7.36 ± 2.40	0.85 ± 0.18	95.08 ± 36.91	6.84 ± 3.10
	A A	10 (2.1)	29.29 ± 10.66	6.39 ± 0.87	8.27 ± 4.82	0.83 ± 0.22	87.64 ± 38.00	6.60 ± 3.31
	A*	114 (12.2)						
***FOXO1***								
rs17446593	A A	372 (78.8)	27.64 ± 8.66	6.32 ± 1.30	7.78 ± 4.20	0.86 ± 0.22	94.27 ± 39.14	6.76 ± 3.27
	A G	93 (19.7)	26.99 ± 7.93	6.44 ± 1.10	8.19 ± 5.19	0.83 ± 0.21	93.14 ± 33.56	6.43 ± 3.06
	G G	7 (1.5)	30.20 ± 9.92	6.56 ± 0.54	12.94 ± 15.44^b^^,^ ^c^	0.75 ± 0.28	84.69 ± 45.78	4.86 ± 2.48
	G*	107 (11.3)						
***FOXO3***								
rs9480865	A A	418 (88.6)	27.63 ± 8.69	6.36 ± 1.31	7.91 ± 4.51	0.85 ± 0.22	94.46 ± 38.64	6.71 ± 3.26
	A G	52 (11.0)	27.25 ± 7.22	6.23 ± 0.72	8.27 ± 6.57	0.86 ± 0.22	91.48 ± 33.45	6.29 ± 3.02
	G G	2 (0.4)	18.64 ± 1.51	5.98 ± 0.05	6.29 ± 0.17	0.96 ± 0.02	51.95 ± 4.60	7.00 ± 0.00
	G*	56 (5.9)						
rs2153960	A A	232 (49.2)	28.36 ± 8.95	6.43 ± 1.28	7.77 ± 4.14	0.86 ± 0.22	98.59 ± 37.96	6.77 ± 3.22
	A G	196 (41.5)	26.29 ± 8.04	6.21 ± 1.27	8.37 ± 5.80	0.84 ± 0.23	88.92 ± 37.02^b^	6.51 ± 3.23
	G G	42 (8.9)	29.30 ± 7.85	6.57 ± 0.95	6.85 ± 1.50	0.91 ± 0.18	93.79 ± 41.52	6.98 ± 3.27
	G*	280 (29.8)						

Data were presented as n (%) for genotypes and alleles or mean±SD for handgrip strength, armGrip, TUG, GS, LPS, and performance score.

*: Minor allele.

^a^: All p-values > 0.05 from Hardy–Weinberg equilibrium test.

^b^: p-value < 0.017 for comparing with AA genotype using Bonferroni test.

^c^: p-value < 0.017 for comparing with AG genotype using Bonferroni test.

### Effect of variation in *IGF-1*, *AKT2*, *FOXO1*, and *FOXO3* on handgrip strength, armGrip, TUG, GS, and LPS

Genotypic and recessive models of handgrip strength, armGrip, TUG, GS, and LPS adjusted for age, gender, BMI, physical activity, smoking, and alcohol intake (adjusted model) are shown in Table 3. The genotype and recessive models showed that risk allele G at *FOXO1* rs17446593 was associated with a higher mean value of TUG (β: 4.07 and β: 4.12, respectively). In the additive and dominant model, differences in the physical performance in four gene polymorphisms were not significant.

**Table 3:** Comparison of handgrip strength, armGrip, TUG, GS, and LPS among the genotype and recessive models.

SNP	Genotype or minor allele	Handgrip strength (kg)		armGrip (kg/kg)		TUG (s)		GS (m/s)		LPS (%)	
		Crude β (SE) ^a^	Adjusted β (SE) ^a^^,^^b^	Crude β (SE) ^a^	Adjusted β (SE) ^a^^,^^b^	Crude β (SE) ^a^	Adjusted β (SE) ^a^^,^^b^	Crude β (SE) ^a^	Adjusted β (SE) ^a^^,^^b^	Crude β (SE) ^a^	Adjusted β (SE) ^a^^,^^b^
**Genotype model**^**c**^											
*IGF-1*											
rs6214	A G	0.59 (0.95)	−0.02 (0.53)	−0.16 (0.14)	−0.21 (0.13)	−0.93 (0.53)	−0.26 (0.48)	0.04 (0.02)	0.01 (0.02)	5.14 (4.44)	1.20 (3.90)
	A A	1.13 (1.11)	0.42 (0.62)	−0.03 (0.16)	−0.08 (0.15)	−1.21 (0.62)	−0.62 (0.55)	0.06 (0.03)*	0.03 (0.02)	4.87 (5.19)	1.42 (4.51)
rs5742692	A G	0.42 (0.83)	0.42 (0.46)	−0.01 (0.12)	−0.04 (0.12)	−0.59 (0.47)	−0.23 (0.42)	0.03 (0.02)	0.01 (0.02)	4.63 (3.90)	3.90 (3.37)
	G G	−0.29 (1.45)	−0.13 (0.80)	0.12 (0.21)	0.05 (0.20)	−1.09 (0.82)	−0.63 (0.72)	0.04 (0.04)	0.02 (0.03)	−1.33 (6.55)	−0.90 (5.66)
rs35767	A G	−0.47 (0.86)	0.56 (0.48)	−0.12 (0.13)	−0.08 (0.12)	−0.14 (0.48)	−0.40 (0.43)	−0.02 (0.02)	0.00 (0.02)	−0.26 (4.03)	2.87 (3.53)
	A A	−0.21 (1.31)	0.89 (0.73)	0.18 (0.19)	0.19 (0.18)	−0.10 (0.73)	−0.36 (0.65)	0.01 (0.03)	0.02 (0.03)	−6.37 (6.02)	−1.44 (5.27)
*AKT2*											
rs892119	A G	0.61 (0.99)	0.22 (0.55)	0.24 (0.15)	0.24 (0.14)	−0.70 (0.56)	−0.46 (0.49)	0.00 (0.03)	−0.01 (0.02)	1.39 (4.64)	0.67 (4.04)
	A A	1.95 (2.73)	0.90 (1.51)	0.09 (0.40)	0.12 (0.38)	0.21 (1.52)	−0.53 (1.35)	−0.02 (0.07)	0.01 (0.06)	−6.06 (12.24)	−2.7 (10.64)
*FOXO1*											
rs17446593	A G	−0.65 (0.99)	−0.07 (0.55)	0.12 (0.15)	0.21 (0.14)	0.42 (0.55)	−0.21 (0.49)	−0.03 (0.03)	0.00 (0.02)	−1.13 (4.63)	2.01 (4.01)
	G G	2.56 (3.26)	1.25 (1.80)	0.24 (0.48)	0.29 (0.45)	5.16 (1.81)*	4.07 (1.59)*	−0.11 (0.08)	−0.07 (0.07)	−9.58 (17.2)	−17.80 (14.93)
*FOXO3*											
rs9480865	A G	−0.38 (1.25)	−0.30 (0.70)	−0.13 (0.18)	−0.03 (0.17)	0.36 (0.71)	0.06 (0.62)	0.00 (0.03)	0.01 (0.03)	−2.98 (5.89)	−2.29 (5.11)
	G G	−9.00 (6.05)	−3.28 (3.35)	−0.38 (0.89)	−0.4 (0.84)	−1.62 (3.38)	−0.16 (2.97)	0.10 (0.16)	0.05 (0.13)	−42.51 (26.99)	−36.33 (23.38)
rs2153960	A G	−2.06 (0.83)*	−0.50 (0.46)	−0.22 (0.12)	−0.11 (0.12)	0.60 (0.47)	0.07 (0.42)	−0.02 (0.02)	0.01 (0.02)	−9.67 (3.88)*	−4.07 (3.40)
	G G	0.94 (1.42)	0.02 (0.79)	0.14 (0.21)	0.09 (0.20)	−0.92 (0.80)	−0.83 (0.70)	0.05 (0.04)	0.04 (0.03)	−4.80 (6.48)	−5.69 (5.64)
**Recessive model**											
*IGF-1*											
rs6214	A	0.75 (0.92)	0.43 (0.51)	0.08 (0.14)	0.06 (0.13)	−0.61 (0.52)	−0.45 (0.46)	0.04 (0.02)	0.02 (0.02)	1.52 (4.31)	0.64 (3.73)
rs5742692	G	−0.47 (1.40)	−0.32 (0.77)	0.13 (0.21)	0.07 (0.19)	−0.83 (0.79)	−0.53 (0.70)	0.03 (0.04)	0.02 (0.03)	−3.38 (6.32)	−2.65 (5.46)
rs35767	A	0.04 (1.23)	0.58 (0.69)	0.24 (0.18)	0.24 (0.17)	−0.02 (0.68)	−0.14 (0.61)	0.02 (0.03)	0.02 (0.03)	−6.24 (5.61)	−2.99 (4.91)
*AKT2*											
rs892119	A	1.82 (2.72)	0.85 (1.51)	0.04 (0.40)	0.08 (0.38)	0.35 (1.52)	−0.44 (1.34)	−0.02 (0.07)	0.01 (0.06)	−6.34 (12.19)	−2.83 (10.60)
*FOXO1*											
rs17446593	G	2.69 (3.25)	1.26 (1.80)	0.22 (0.48)	0.25 (0.45)	5.08 (1.80)*	4.12 (1.58)*	−0.11 (0.08)	−0.07 (0.07)	−9.35 (17.15)	−18.17 (14.9)
*FOXO3*											
rs9480865	G	−8.96 (6.04)	−3.25 (3.35)	−0.37 (0.89)	−0.40 (0.84)	−1.66 (3.38)	−0.17 (2.97)	0.10 (0.16)	0.05 (0.13)	−42.18 (26.96)	−36.11 (23.35)
rs2153960	G	1.89 (1.38)	0.24 (0.77)	0.24 (0.20)	0.14 (0.19)	−1.20 (0.77)	−0.86 (0.68)	0.06 (0.04)	0.04 (0.03)	−0.33 (6.27)	−3.87 (5.44)

^a^: β is estimated coefficient of regression model and SE is standard error.

^b^: Adjustment for age, sex, BMI, physical activity, smoking status, and alcohol drinking.

^c^: Using major/major genotype as reference genotype in genotype model.

*: p-value < 0.05.

### Gene–gene and gene–physical activity interactions on armGrip, TUG, LPS, and GS

Before performing FDR adjusted, we found the following significant gene–gene interactions on physical performances: the interactions between *IGF-1* SNP rs6214, *FOXO1* SNP rs17446593, and *FOXO3* SNP rs2153960 for TUG (P = 0.005 and 0.032, respectively); the interactions of *IGF-1* SNP rs5742692 with *FOXO3* SNP rs9480865 on LPS (P = 0.018) and with rs2153960 on armGrip (P = 0.012); similarly, the interaction between *IGF-1* SNP rs35767 and *FOXO3* SNP rs2153960 on armGrip (P = 0.035). However, these significant interactions became borderline significant or insignificant after FDR adjustment.

**Fig 1:**
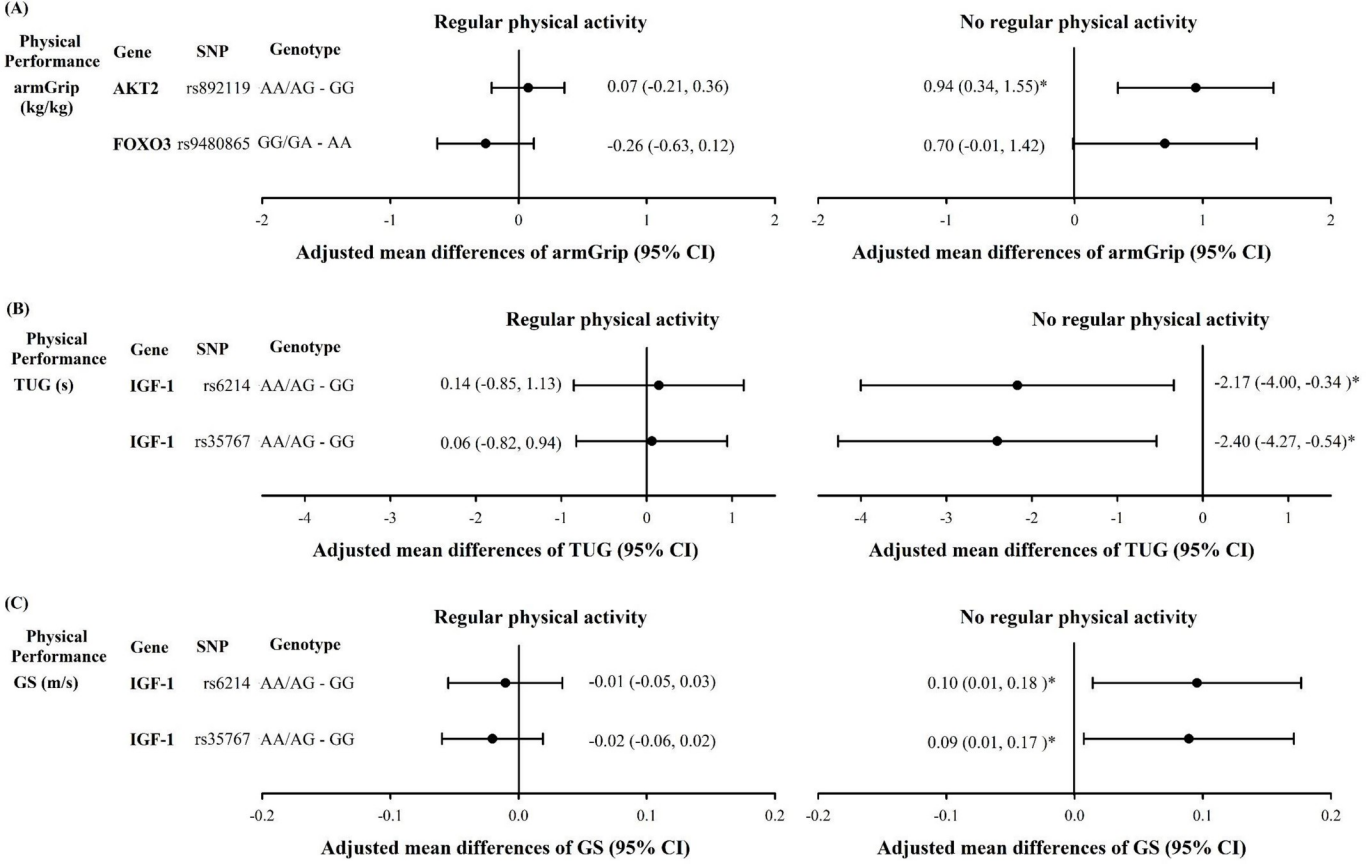
Adjusted mean differences of armGrip (A), TUG (B), and GS (C) in *IGF-1*, *AKT2*, and *FOXO3* SNPs stratified by physical activity for significant gene–physical activity interactions. Additionally adjustment of age, sex, BMI, smoking status, and alcohol drinking. *: p<0.05.

The significant gene–physical activity interactions after FDR adjustment are summarized in Table 4. For TUG and GS, the interactions of *IGF-1* SNP rs6214 (P = 0.045 and 0.038, respectively) and rs35767 (P = 0.045 and 0.038, respectively) with regular physical activity were significant. The interactions between *AKT2* SNP rs892119 and *FOXO3* SNP rs9480865 with regular physical activity on armGrip were also significant (P = 0.011 and 0.040, respectively). For the gene–physical activity interaction, among elders with no regular physical activity, the A genotype carriers at *AKT2* rs892119 had higher armGrip measures than those with the GG genotype (0.94, 95% CI: 0.34, 1.55) (Fig 1A). Among elders with no regular physical activity, the A genotype carriers at *IGF-1* rs6214 and rs35767 had lower TUG measures than those with the GG genotype (−2.17, 95% CI: −4.00, −0.34; −2.40, 95% CI: −4.27, −0.54, respectively) (Fig 1B). The GS of inactive elderly carrying AA/AG genotype of *IGF-1* rs6214 or rs35767 was higher than that of elders with GG genotype (0.10, 95% CI: 0.01, 0.18; 0.09, 95% CI: 0.01, 0.17, respectively) (Fig 1C).

**Table 4:** Significant gene–physical activity interactions on armGrip, TUG, and GS.

Gene	SNP	P for interaction^a^		
		armGrip	TUG	GS
*IGF-1*	rs6214		0.045	0.038
*IGF-1*	rs35767		0.045	0.038
*AKT2*	rs892119	0.011		
*FOXO3*	rs9480865	0.040		

^a^: FDR p-value. Additionally adjustment of age, sex, BMI, smoking status, and alcohol drinking.

## Discussion

The IGF1–AKT–FOXO pathway may play an important role in aging and incident functional disability [18]. Physical performance has also been correlated with physical activity levels and functional decline risks in the elderly population [2, 3, 15, 16]. This study is the first to report the correlation between the variations in *IGF-1*, *AKT2*, *FOXO1*, and *FOXO3* and the physical performance in community-dwelling elderly subjects in Taiwan. We investigated the associations of physical performance with gene–gene interactions and explored the interaction effect of gene and physical activity. For gene–physical activity interaction, physically inactive elders with the AA/AG genotype of *AKT2* rs892119 had high adjusted mean differences in armGrip. Similarly, inactive individuals carrying the AA/AG genotype of *IGF-1* rs6214 or rs35767 had low adjusted mean differences in TUG and a high GS.

The minor allele frequencies (MAFs) of the SNPs in our subjects were distributed similarly to those of the Han Chinese in China (CHB) (data available from the International HapMap Project, http://hapmap.ncbi.nlm.nih.gov; Data Rel 27 Phase II+III, Feb 09). Our study also analyzed the pairwise relation among seven variants by using the genotypic data from 472 unrelated elders. LD between SNPs with moderate allelic correlation was observed between *IGF-1* rs5742692 and rs35767 variants (*r*^*2*^ = 0.48), and weak LD existed between *FOXO3* rs9480865 and rs2153960 (*r*^*2*^ = 0.12).

Two common SNPs in *IGF-1* (rs35767 and rs972936) can affect the circulating IGF-1 level [22, 51]. IGF-1 concentration progressively decreases with age [52]. For example, circulating IGF-1 levels decrease with age at a rate of 1.7 ng/mL per year in individuals older than 50 years [53]. Low IGF-1 levels in older women are associated with poor muscle strength, slow walking speed, and difficulty in performing mobility tasks [23]. Our previous study reported elders carrying G allele of rs6214 on *IGF-1* are significantly correlated with lower serum IGF-1 levels and serum IGF-1 level of the low appendicular skeletal muscle mass index (ASMI) group is significantly lower than that of the normal ASMI group [54]. Alfred et al. [55] reported SNP rs35767 of *IGF-1* is not correlated with physical performance, and this observation is consistent with our findings, which revealed the main effects of three common genetic variants (rs6214, rs5742692, and rs35767 SNPs) of *IGF-1* were unrelated to physical performance of elderly persons.

A previous study identified the main effect of *IGF-1* (SNP rs35767) on endurance performance in athletes [56]. Because this SNP rs35767 is located in the promoter region of the *IGF-1* gene, it may influence the gene expression of *IGF-1*, and then affect physical performance. Additionally, another study pointed out that muscle strength was influenced by the polymorphisms in the promoter region of *IGF-1* after strength training in older adults [57]. These two prior studies demonstrated the main effect of IGF-1 on physical performance and our study provided further evidence that physical performances of the elderly were affected by the interaction between polymorphism of IGF-1 and regular physical activity. The inactive elders who carried the GG genotype of rs6214 or rs35767 had worse TUG and GS. On the contrary, among the elderly with regular physical activity habits, no matter what genotypes they carried, there were no significant differences in their physical performances. Therefore, exercise programs should target the elderly to develop a habit of regular physical activity, especially those with the GG genotype of rs6214 or rs35767 of *IGF-1* to prevent poor physical performance.

*AKT2* is one of three isoforms of AKTs (*AKT1*, *AKT2*, and *AKT3*) [58]. In a mouse experiment, AKT has been genetically disrupted express growth defects [59], and *AKT2* is disrupted suffer skeletal muscle atrophy [60]. However, our study indicated no association between SNPs in *AKT2* and physical performance was observed but SNPs in *AKT2* interacted with physical activity in the elderly.

Mammalian skeletal muscle cells contain three isoforms of the FOXO family, namely, *FOXO1*, *FOXO3*, and *FOXO4* [61]. Transgenic expression of *FOXO1* in skeletal muscle mass causes a marked decrease in muscle mass and fiber atrophy in transgenic mice [35]. *FOXO3* promotes atrophy-related gene expression and muscle atrophy in vivo. We found that the risk allele G at *FOXO1* rs17446593 is associated with an increased risk of slow TUG among the elderly. In terms of armGrip, although the interaction of *FOXO3* rs9480865 and physical activity was significant, it turned into non-significant in the stratification analysis which was stratified by physical activity. This possible explanation is our sample size was not large enough because *FOXO3* rs9480865was borderline significant (P = 0.0537, Fig 1(A)) among elders with no regular physical activity.

Our study has two potential limitations. First, our findings should be carefully interpreted because of the relatively small sample size. The number of individuals who underwent physical performance measurements might be too small to provide sufficient statistical power for stratified analysis. However, significant gene–physical activity interactions were detected even with a small sample and limited power. Future research to examine this issue using a larger sample would provide more precise estimates that could help lead to firmer conclusions regarding the role of interactions among *IGF-1*, *AKT2*, *FOXO1*, and *FOXO3* variations and between genes and physical activities on physical performance. Study samples recruited from a well-defined geographical area could improve the representativeness of older people. Second, we searched for candidate variants in relevant literature to assess their effects. Our observed effect might be due to high LD with a true susceptibility allele. Future studies may involve the sequencing of these genes to further answer these questions.

## Conclusion

This study presented evidence supporting the interactive effects of *IGF-1*, *AKT2*, and *FOXO3* with physical inactivity on poor physical performance, suggesting that physical activity might modulate the effects of genotypes on physical performance. These findings could reveal the genetic mechanisms underlying physical performance. A sedentary lifestyle may increase the risk of impairing physical performance and regular physical activity is a remedy for sarcopenia, even a little regular physical activity can overcome carrying some risk alleles in this pathway.
